# Wet chemical synthesis of nanocrystalline hydroxyapatite flakes: effect of pH and sintering temperature on structural and morphological properties

**DOI:** 10.1098/rsos.180962

**Published:** 2018-08-15

**Authors:** V. Rodríguez-Lugo, T. V. K. Karthik, D. Mendoza-Anaya, E. Rubio-Rosas, L. S. Villaseñor Cerón, M. I. Reyes-Valderrama, E. Salinas-Rodríguez

**Affiliations:** 1 Área Académica de Ciencias de la Tierra y Materiales, Universidad Autónoma del Estado de Hidalgo, Carr. Pachuca—Tulancingo km 4.5, C.P. 42184 Pachuca, Hidalgo, México; 2 Instituto Nacional de Investigaciones Nucleares, Carr. México-Toluca S/N, La Marquesa, Ocoyoacac, Edo. de México C.P. 52750, México; 3 Benemérita Universidad Autónoma de Puebla, Prolongación de la 24 Sur y San Claudio, Ciudad Universitaria, Sn Manuel, 72570 Puebla, México

**Keywords:** hydroxyapatite, pH, hydrothermal, vibrational modes, crystallite size

## Abstract

Wet chemical synthesis of hydroxyapatite (HAp) nanostructures was carried out with different solution pH values (9, 10 and 11) and sintering temperatures (300°C, 500°C, 700°C and 900°C). The effects of pH and sintering temperature on the structural and morphological properties of nanocrystalline HAp powders were presented. Fourier transform infrared spectroscopy (FTIR), X-ray diffraction (XRD) and scanning electron microscopy (SEM) analysis were performed to obtain the crystalline structure, chemical composition, morphology and particle size of the HAp powders. The TEM analysis is used in order to observe the rod- and flake-like HAp structures. XRD confirms the presence of both HAp hexagonal and monetite phases, although the monetite phase was less abundant in the resultant powders. Increase in pH reduced the monetite phase and enhanced Ca/P ratio from 1.7 to 1.83. Additionally, an increment in sintering temperature increased the crystallite size from 20 to 56 nm. The SEM analysis revealed the formation of semi-spherical and flake-like HAp structures with preferential flake morphology. An increase in pH and sintering temperature resulted in the growth and coalescence of crystals resulting in a porous capsular morphology. The FTIR analysis confirmed the reduction of carbonate stretching modes with an increase in pH and H–O–H antisymmetric stretching mode is eliminated for powders sintered at 900°C confirming the formation of stable and porous HAp powders.

## 1. Introduction

Currently, the biomaterial technologies are widely used in the healthcare industry, cell biology and drug delivery systems [1]. Furthermore, the research in this field is continually under development to meet the future needs of the market, mostly in bioimplants [2–4], which makes the innovation of new biomaterial synthesis extremely important. One of the most relevant ceramic biomaterials from the calcium phosphate family is hydroxyapatite (HAp), which is widely used in the fabrication of implants due to its high biocompatibility with soft tissues, muscles and skin. Additionally, its similar composition to minerals in the human bones and teeth makes the union simple and effective with the bones in a natural way [5]. A great and dedicated effort has also been made to control the shape, grain size, chemical purity and crystalline degree of HAp through several alternative routes [6–12].

One of the most used methods to obtain synthetic HAp is the ‘conventional solid-state method’, in which the particles obtained maintain the shape of the phosphate precursor particles [5]. On the other hand, the ‘wet chemical method’ is performed using aqueous solutions at relatively low temperatures like those in living beings [13], and the particles obtained by this method usually are porous and exhibit an inhomogeneous chemical composition [14,15]. But in the ‘hydrolysis reaction’ method, the chemical reaction of calcium phosphate with water is performed at 100°C [16]. Moreover, the ‘hydrothermal method’, which includes chemical reactions of aqueous solutions with the precursor materials, allows obtaining well-crystallized powders with homogeneous chemical composition [17–23]. Besides the works mentioned above, there are many other chemical synthesis methods for preparing micro- and nano-HAp structures [24–28], but the results show that there are still serious difficulties to control the particle size and crystalline degree of HAp. In addition, the final performance of the biomaterials is degraded by the secondary products of the chemical reactions and/or unreacted species during the synthesis. Physical methods to synthesize biomaterials, such as selective laser sintering, present similar difficulties to obtain a final product with desirable characteristics [29,30].

Recently, there has been a great demand for large quantities of inexpensive HAp nanoparticles with well-defined morphology and crystalline phase for diverse commercial applications such as three-dimensional printing. In this regard, the wet chemical precipitation technique has various advantages such as simplicity, low reaction time and temperatures, high purity and cost-effectiveness. Principally, scalability and reproducibility using wet chemical process are potential [24,27,31].

The best way to obtain a synthetic biomaterial with desirable characteristics is to keep control of the synthesis parameters like precursor concentration, sintering time, temperature, acidity (or pH) and volume of the solvent. In general, improvement of the bioactivity and biocompatibility is related to a smaller HAp particle size with a stoichiometric Ca/P ratio (approx. 1.6). This can be achieved by varying pH and the sintering process which subsequently control the shape and size distribution of the resulting biomaterials [18,20,32,33]. In this work, the wet precipitation method is used to obtain homogeneous and porous HAp powders with nano- and micrometre structures with ammonium hydroxide as precipitation agent by varying pH and the sintering temperature. A detailed discussion about the change in morphology, chemical structure (functional groups), Ca/P ratio and crystallite size by using combined precipitation–sintering method is presented.

## 2. Material and methods

### 2.1. Synthesis of hydroxyapatite powders

All the chemicals used in this work were purchased from Sigma Aldrich with high purity (greater than 99%). Calcium nitrate [Ca(NO_3_)_2_] and ammonium phosphate [(NH_4_)_3_PO_4_] were used as precursors for obtaining HAp and ammonium hydroxide (NH_4_OH) as a precipitation agent.

Primarily, aqueous 0.042 M of Ca(NO_3_)_2_ and 0.025 M of (NH_4_)_3_PO_4_ solutions were prepared separately. Subsequently, previously prepared Ca(NO_3_)_2_ solution equipped with rosary-type condenser in the central neck in a 500 ml three-neck round-bottom flask was transferred to a silicon oil thermal bath maintained at 90°C with a magnetic stirrer. Aqueous (NH_4_)_3_PO_4_ solution followed by NH_4_OH solution was added dropwise through the left neck of the flask by monitoring the pH with a potentiometer placed in the right neck of the flask. After the desired pH had been achieved, the solution was left stirring for 3 h to obtain the HAp powders. The chemical precipitation reaction for the synthesis of HAp is shown in equation (2.1) [34]. The procedure was replicated for each pH value. Finally, all the HAp powders obtained at different pH values (9, 10 and 11) were sintered at 300°C, 500°C, 700°C and 900°C for 1 h, with a heating rate of 1°C min^−1^.

2.1 $$\text{10Ca}{\left(\text{N}{\text{O}}_{3}\right)}_{2}\cdot 4{\text{H}}_{2}\text{O + 6}{\left(\text{N}{\text{H}}_{4}\right)}_{2}\text{HP}{\text{O}}_{4}+8N{\text{H}}_{4}\text{OH}\to \text{C}{\text{a}}_{10}{\left(\text{P}{\text{O}}_{4}\right)}_{6}{\left(\text{OH}\right)}_{2}+20N{\text{H}}_{4}\text{N}{\text{O}}_{3+}\;46{\text{H}}_{2}\text{O}.$$

### 2.2. Materials’ characterization

The morphological characterization of the HAp powders was performed using low-vacuum scanning electron microscopy (SEM, JEOL JSM5900-LV) equipped with an Oxford energy-dispersive X-ray spectroscope (EDS), operated at 20 kV. The X-ray diffraction analysis was carried out by a Siemens D5000 diffractometer powder technique in the range of 20°–60° in 2*θ* (°) to identify the crystalline phases. To identify the functional groups in HAp samples, an infrared spectroscopy analysis was performed using Nicolet Nexus 670 FTIR operated at room conditions in the range of 4000–400 cm^−1^.

## 3. Results

### 3.1. Scanning electron microscopy and energy-dispersive X-ray spectroscopy analysis

**Figure 1.**
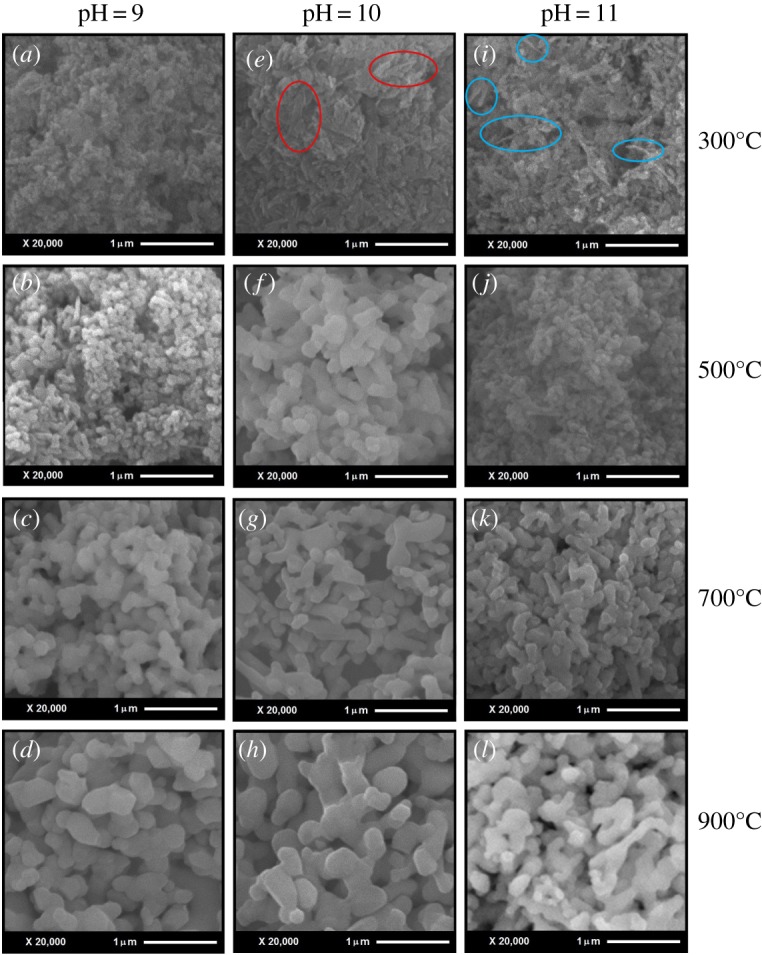
SEM images of the HAp powders sintered at 300°C, 500°C, 700°C and 900°C with pH 9 (*a–d*), 10 (*e–h*) and 11 (*i–l*).

SEM was employed to obtain the morphological characteristics of the HAp powders. Figure 1 shows the SEM images of the HAp powders sintered at 300°C, 500°C, 700°C and 900°C at pH values 9 (figure 1*a–d*), 10 (figure 1*e–h*) and 11 (figure 1*i–l*).

**Figure 2.**
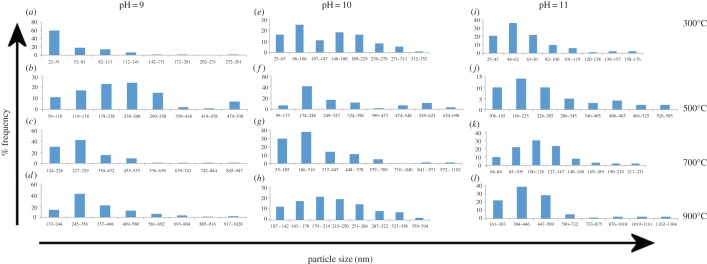
Histograms of the obtained particle size distribution of the HAp powders sintered at 300°C, 500°C, 700°C and 900°C with pH 9 (*a–d*), 10 (*e–h*) and 11 (*i–l*).

**Figure 3.**
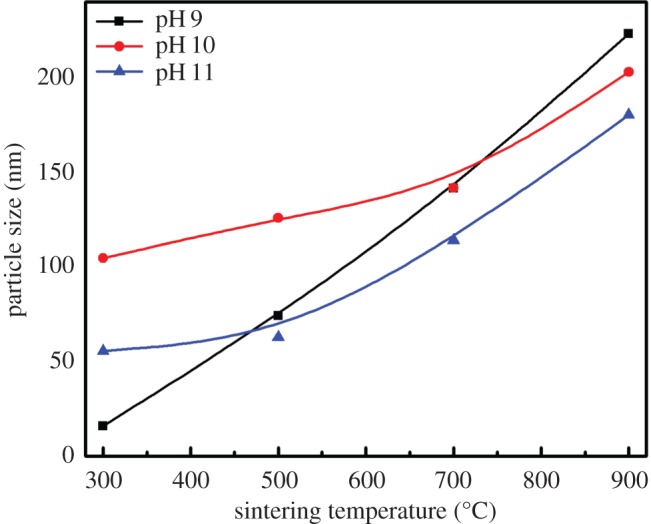
Average particle size of HAp powders for different temperatures and pH.

**Figure 4.**
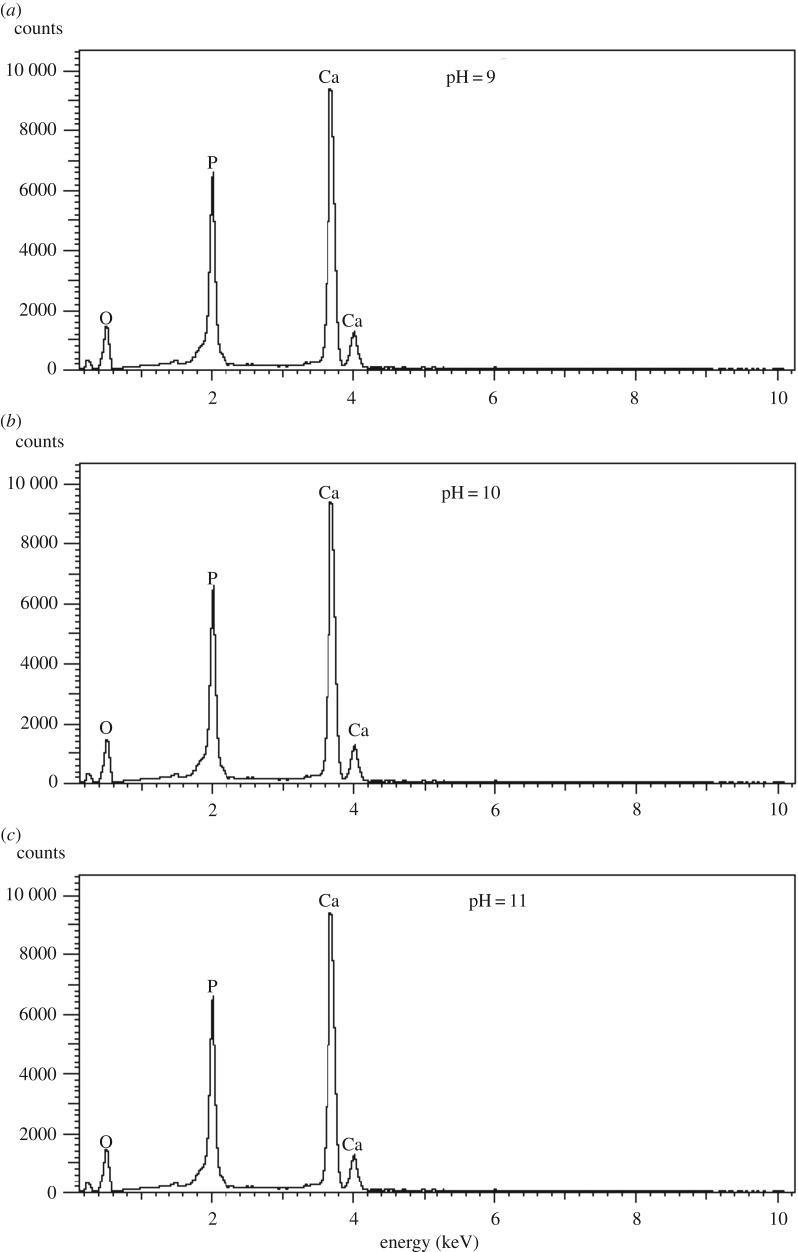
EDS spectra of the HAp powders calcinated at 900°C for pH values (*a*) 9, (*b*) 10 and (*c*) 11.

SEM micrographs show an important influence of the sintering temperature on the morphology of HAp. Increase in sintering temperature changed completely the HAp morphology from semi-spherical nanoparticles to flake-like structures. As the sintering temperature increases, the evaporation of the residues makes the morphology as an interconnected flake-like porous network (figure 1*d,h,l*). Figures 2 and 3 show the particle size histograms and the variation of particle size with respect to the sintering temperature. From figures 3 and 4, it is evident that the increase in the sintering temperature induced the growth of the particle size from tens of nanometres to some micrometres for all pH values. This may be due to the diffusion of the particles at higher temperatures and formation of flake-like structures with interconnections [35,36].

Apparent influence of the pH on the shape of the HAp agglomerates is revealed by a careful comparison of figure 1*a,e,i*, in which semi-spherical particles ranging from 30 to 50 nm, rod-like structures approximately 40–80 nm and flake-like structures approximately 80–150 nm are observed. Also, for pH 10, the formation of interconnections started at lower temperatures of approximately 500°C (figure 1*f*), whereas for pH 9 and 11, the formation of interconnections started at 700°C (figure 1*c,k*). In order to calculate the Ca/P ratios, the EDS analysis of all the samples was performed and figure 4*a–c* shows the EDS spectra of HAp powders calcinated at 900°C for pH 9, 10 and 11. The Ca/P ratios obtained for all samples using the EDS analysis were from 1.66 to 1.83 and are presented in table 1. For being used in bioactivity, a pure stoichiometric HAp powder Ca/P ratio should be 1.67 [37,38] but only the samples synthesized at pH 10 possess the Ca/P ratio close to the stoichiometric HAp. The detailed growth mechanism is explained in §3.5 relating XRD and FTIR results.

**Table 1.** Ca/P ratios of HAp powders for different temperatures and pH values.

temperature (°C)	Ca/P ratio		
	pH = 9	pH = 10	pH = 11
as prepared	1.70	1.66	1.74
300	1.71	1.67	1.76
500	1.75	1.72	1.80
700	1.79	1.74	1.81
900	1.81	1.79	1.83

### 3.2. Transmission electron microscopy analysis

**Figure 5.**
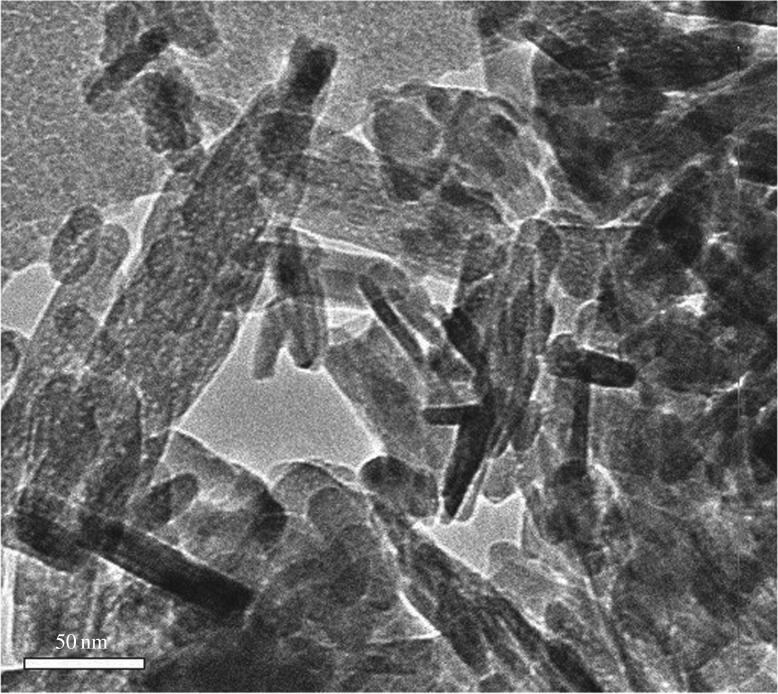
TEM image of the HAp powders sintered at 300°C with pH value 11.

Transmission electron microscopy (TEM) was employed to observe the rod-like structures that were observed in the SEM analysis for HAp powders calcinated at 300°C. Figure 5 shows the TEM images of the HAp powders sintered at 300°C and at pH 11. Detailed TEM analysis was impossible due to the agglomeration of the HAp structures. From figure 5, it is observed that the powders contain a mixture of semi-spherical, rod- and flake-like morphological structures. The rod-like morphology is predominant compared to remaining structures. Owing to the overlapping of the structures, it is difficult to analyse the exact structure of the rods, but a minimum diameter of approximately 20 nm and a minimum length of approximately 40 nm were observed.

### 3.3. X-ray diffraction analysis

**Figure 6.**
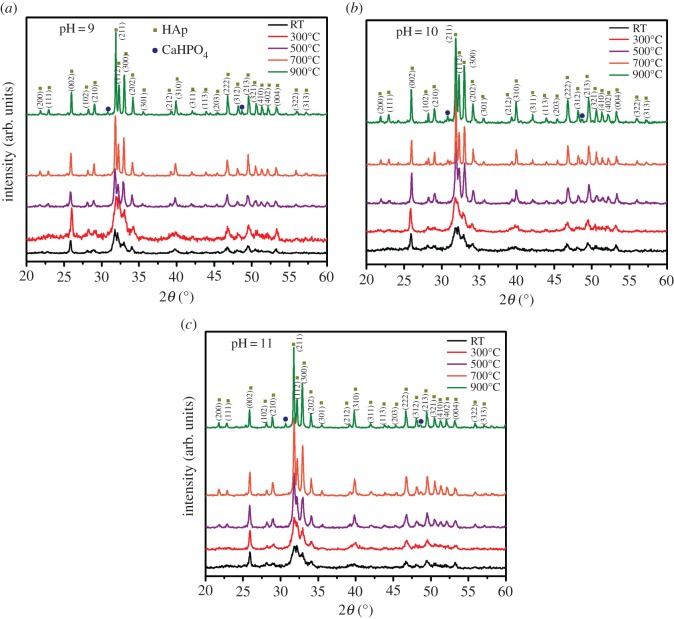
XRD images of the HAp powders with pH (*a*) 9, (*b*) 10, (*c*) 11 and different sintering temperatures.

The XRD patterns of as-prepared and sintered HAp powders obtained at pH 9, 10 and 11 are shown in figure 6*a–c*, respectively. A glance at the XRD spectra shows the formation of two crystalline phases of calcium phosphates: hydroxyapatite (HAp) and monetite (CaHPO_4_) with a preferential orientation in (211) plane. The main diffraction peaks in this pattern match with two JCPDS cards: no. 46-0905 and no. 09-0080, corresponding to HAp and monetite, respectively [13,39]. The diffraction peaks at 25.88°, 31.76°, 32.16°, 32.92° and 39.8° in 2*θ* (°) correspond to (002), (211), (112), (300) and (311) planes for HAp, respectively. Diffraction peaks located at 30.68° and 48.6° in 2*θ* correspond to the monetite phase.

**Figure 7.**
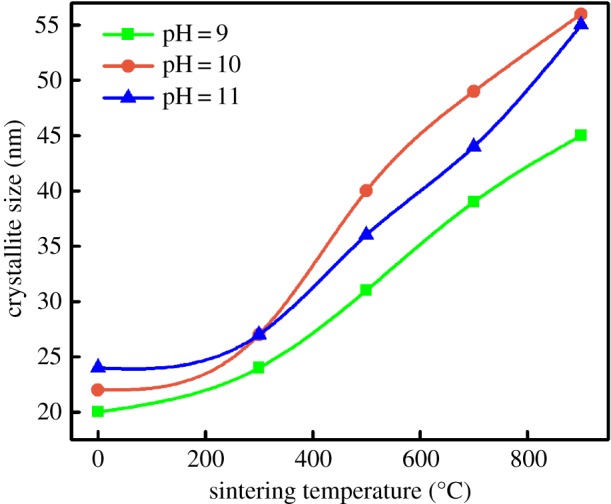
Crystallite size of HAp powders for different temperatures and pH.

The crystallite size for HAp was calculated from X-ray diffractogram of strong reflections with intensity % by measuring the full width at half maximum (FWHM). The Scherrer equation for calculating the crystallite size is given as follows:

3.1 $$D=\frac{K\lambda }{\beta_{hkl}.\text{cos}\theta_{hkl}},$$

where *K* is the Scherrer constant, *λ* is the wavelength of X-ray for diffraction, *β_hkl_* is the FWHM of the corresponding plane and *θ_hkl_* is the angle measured of the same plane [13,40]. The constant *K* in the above formula generally takes the value 0.9, assuming the shape of crystallites. According to the Scherrer equation, the crystallite size obtained for HAp was in the range of 20–56 nm (figure 7).

After comparing all the XRD patterns of figure 6, it is observed that all the diffraction peaks were more narrow with the increase in the sintering temperature. This implies that the samples improved their crystallinity and crystallite size (figure 7) due to the reduction in strain and defect density of the crystals. The presence of CaHPO_4_ was observed only for samples sintered at 700°C and 900°C, and it is due to the increase in the number of chemical reactions between the residual species [41], which resulted in agglomeration and formation of new compounds [42,43].

By comparing figures 6 and 7, it is observed that for the samples with pH 10, the crystallite sizes were higher for samples sintered at 500°C, 700°C and 900°C. It is possible to note that the intense peak at 32.92° in 2*θ*, which is associated with (300) plane for HAp is lower. As explained in §3.1, release of Ca^2+^, PO_4_^3−^ and OH^−^ ions restrict the growth in (300) plane [44].

We believe that the presence of adequate OH^−^ ions for the pH 10 resulted in optimum Ca/P ratio (table 1) which subsequently improved the HAp plane, for example (211) and (300), and reduced the monetite peak intensities compared to other pH values. Therefore, from XRD and SEM analyses, it can be reasoned out that the pH 10 is optimum to obtain more pure HAp with desirable crystallite size and interconnected flake morphology for biomedical applications [45–48].

### 3.4. Fourier transform infrared spectroscopy analysis

**Figure 8.**
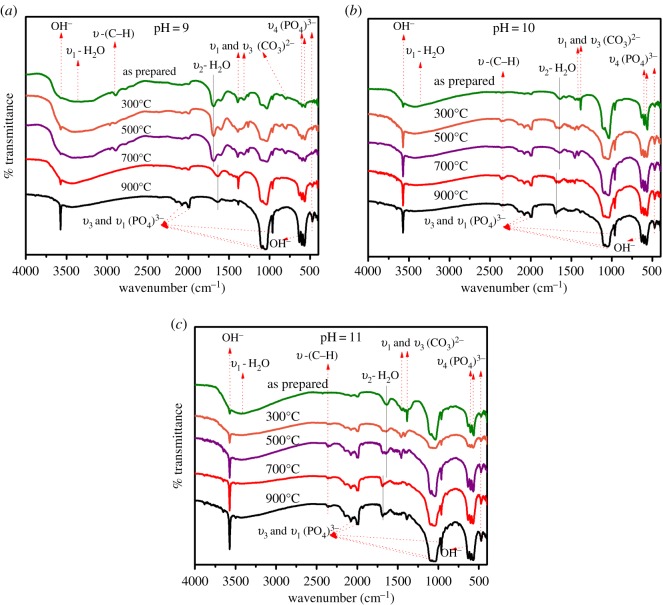
FTIR spectra of the HAp powders with pH (*a*) 9, (*b*) 10, (*c*) 11 and different sintering temperatures.

Figure 8*a–c* shows the FTIR spectra of the HAp powders at pH 9, 10 and 11 for different sintering temperatures. Depending on the pH and sintering temperatures, approximately 16 different absorption bands corresponding to several vibrational modes of functional groups were identified [49,50] and are tabulated in table 2. In accordance with figure 8 and table 2, we can identify the presence of phosphate (PO_4_^3−^), hydroxyl (OH^−^) and carbonate (CO_3_^2−^), and water molecule (H_2_O) ions and their corresponding bending and stretching modes. Then, IR spectroscopy confirmed the formation of HAp under different pH and sintering temperatures. The absence of nitrogen and nitrate group bands evidences a complete consumption of the precursor reagents and an absolute chemical synthesis was performed.

**Table 2.** The IR bands observed in HAp samples prepared at different pH values and sintering temperature.

vibrational modes	FTIR active bands at wavenumber (cm^−1^)		
	pH = 9	pH = 10	pH = 11
*ν*_2_ symmetric bending mode of O–P–O in phosphate ions	469	469	469
*ν*_4_ symmetric bending mode of O–P–O in phosphate ions	567	567	567
*ν*_4_ symmetric bending mode of O–P–O in phosphate ions	603	603	603
symmetric stretching mode of hydroxyl ions	629	629	629
*ν*_2_ of carbonate ions	873	—	—
*ν*_1_ symmetric stretching mode of P–O in phosphate ions	961	961	961
*ν*_3_ asymmetric stretching mode of P–O in phosphate ions	1041	1041	1041
*ν*_3_ asymmetric stretching mode of P–O in phosphate ions	1091	1091	1091
*ν*_1_ symmetric stretching of carbonate ions	1321	1321	1321
*ν*_3_ of carbonate ions	1417	1417	1417
*ν*_2_ bending mode of H–O–H in lattice H_2_O	1630 and 1650	1630 and 1650	1630 and 1650
*ν*_3_ asymmetric stretching mode of P–O in phosphate ions	2002	2002	2002
*ν*_1_ asymmetric stretching mode of P–O in phosphate ions	2072	2072	2072
stretching mode of C–H	—	2390	2390
stretching mode of C–H	2901	—	—
*ν*_1_ stretching mode of lattice H_2_O	3464	3464	3464
symmetric stretching mode of hydroxyl ions	3570	3570	3570

Phosphate ion vibrational modes are observed at 469, 567 and 603 cm^−1^ which correspond to *ν*_2_ and *ν*_4_ symmetric bending mode of O–P–O in phosphate ions. Bands at 961, 1041 and 1091 cm^−1^ are of *ν*_1_-symmetric and *ν*_3_-asymmetric stretching modes of P–O in phosphate ions. The group of bands at 2002 and 2072 cm^−1^ corresponds to *ν*_1_ and *ν*_3_-asymmetric stretching modes of phosphate ions.

All the above-mentioned phosphate bands are observed at all pH values, but these bands become stronger and narrow with the increasing sintering temperature, which confirms the growth of phosphate ions and crystallization of HAp at higher temperatures.

Additionally, two pronounced peaks in the IR spectra at 629 and 3570 cm^−1^ were identified and associated with the symmetric stretching mode of hydroxyl group. The hydroxyl stretching modes at 629 and 3570 cm^−1^ become available continuously with the increase in temperature from room temperature to 900°C, showing that HAp was obtained from the beginning; sintering temperature only modified the crystalline degree of HAp, according to XRD patterns (§3.3). The OH^−^ band at 3570 cm^−1^ shows a weak intensity for samples at pH 9 and goes on improving from samples at pH 10 and 11, which makes it clear that the amount of OH^−^ ions present is increasing for higher pH values; this result is in good agreement with the SEM and XRD results. As the pH of the solution increases, the presence of the OH^−^ ions also increases and this leads to the growth in desired planes like (300) and (211) (XRD results) which subsequently resulted in structures with flake- and rod-like morphology (SEM results).

The intensity bands observed at 873, 1321 and 1417 cm^−1^ in the IR spectrum are attributed to components of *ν*_2_, *ν*_1_ and *ν*_3_ symmetric modes of carbonate ions. The carbonate mode at 873 cm^−1^ is only visible for samples at pH 9, and it disappears for samples at pH 10 and 11. However, carbonate bands at 1321 and 1417 cm^−1^ are present for all pH values. An ideal and pure HAp structure should not present any carbonate vibrational modes, but its presence at 1321 and 1417 cm^−1^ may be due to its atmospheric adsorption after the synthesis, which is unavoidable in chemical synthesis techniques, whereas the presence of carbonate band at 873 could be associated with the synthesis product but it diminishes by increasing the pH. Increasing of the sintering temperature also diminishes the presence of the carbonate band intensities, which is in good agreement with XRD and SEM results.

The broad envelope observed in all HAp powders in the IR spectra at 3464 cm^−1^ is assigned to *ν*_1_-stretching mode of water (H_2_O) molecule and its intensity decreases with the increase in sintering temperature, and this peak is eliminated at 900°C, which indicates the evaporation of the solvents during the sintering process. Also, two bands at 1630 and 1650 cm^−1^ correspond to *ν*_2_ bending modes of H–O–H in H_2_O lattice (figure 8 and table 2). At pH value 9 (figure 8*a*), the 1650 cm^−1^ band appears only for as-prepared samples and sintered for 300°C and 500°C. But, for samples sintered at 700°C and 900°C, 1650 cm^−1^ band vanishes and a band at 1630 cm^−1^ appears. A contrary behaviour is observed for samples with pH values 10 and 11 (figure 8*b,c*). This transition of water molecule bending modes at temperature 700°C and at pH 10 resulted in optimum Ca/P values (table 1) for obtaining pure and stoichiometric HAp powders. The presence of small peaks at 2901 cm^−1^ for samples with pH 9 (figure 8*a*) and at 2390 cm^−1^ for samples with pH 10 and 11 and are attributed to the C–H stretching modes originated due to the presence of the monetite phase, which is also confirmed in the XRD analysis. We believe that the presence of monetite peaks is corroborated by the partial decomposition of the HAp phase due to its sintering in air [51,52].

### 3.5. Discussion

As the solution pH is increased by the addition of NH_4_OH, the Ca^2+^, (PO_4_)^3−^ and OH^−^ ions are liberated and the OH^−^ ions are situated on the facets of the formed nuclei [44,53]. Later, the growth occurs only on the planes without OH^−^ ions on their surface. For pH 9, semi-spherical particle with deviated Ca/P ratio is obtained (figure 1*a*) and when the pH is increased to 10, OH^−^ ion concentration also increases on the facets, which restricts the growth in that facet and allows the growth in the facets without OH^−^ ions, which is the preferential orientation. This resulted in the flake-like structures with adequate Ca/P ratio (marked in red circles in figure 1*e*).

Furthermore, with the increase in the pH to 11, Ca/P ratio increases and restricts the growth due to the presence of more OH^−^ ions, which resulted in rod-like structure (marked with blue circles in figure 1*i*). Therefore, increased pH restricted the growth in some planes and resulted in flake- and rod-like HAp nanostructures. Additionally, increasing pH value improved the solubility which subsequently fastened the precipitation and agglomeration of particles [54–56]. Thus, pH is an important parameter to acquire the desired morphology.

**Figure 9.**
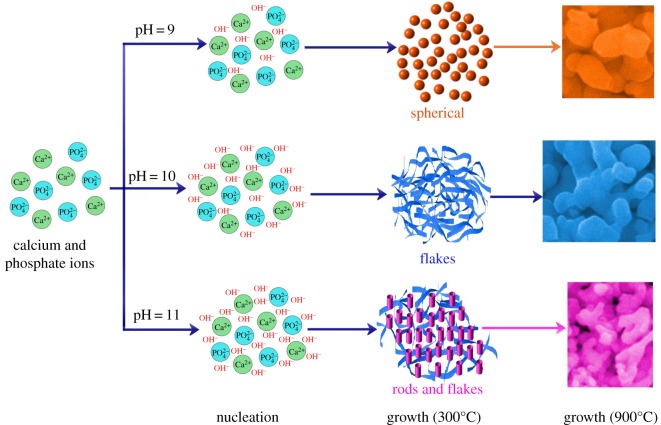
Morphological transformation of the HAp powders with the increase in pH and sintering temperature.

Figure 9 shows the morphological transformation of synthesized HAp particles with respect to the pH and sintering temperature. It is evident from figure 9 that the increase in pH affected the HAp morphology, whereas increase in the sintering temperature resulted in coalescence of particles.

Another key factor that is affected due to the change in pH and sintering temperature is Ca/P ratio (amount of calcium and phosphate phases) of HAp. For a stoichiometric HAp powder, Ca/P ratio is 1.67, which is observed for samples prepared with pH 10 and sintered at 300°C and 500°C. But XRD confirms a mixture of both HAp and monetite phases, which is like commercially available HAp with identical XRD spectrum of stoichiometric HAp (Ca/P = 1.67) [51,57–60]. We believe that the presence of adequate OH^−^ ions for the pH 10 resulted in optimum Ca/P ratio (table 1) which subsequently improved the HAp plane, for example (211) and (300), and reduced the monetite peak intensities compared to other pH values.

Therefore, based on FTIR, SEM and XRD analyses, we can say that the pH and the sintering temperature play an important role on the purity, morphology and the crystallinity of the HAp structures and also it can be reasoned out that the pH 10 is optimum to obtain relatively stoichiometric HAp with desirable crystallite size and interconnected flake morphology for biomedical applications [45–48]. As we mentioned previously in the Introduction section, HAp obtained in this work can be used as an implantation material, to repair injured bones and teeth. However, before being used as biocompatible material, the HAp must have satisfied requirements associated with the physicochemical characteristics such as shape, size, chemical purity and crystalline degree. Therefore, our methodology is relevant to obtain HAp with size- and shape-controlled nanostructures by varying pH or sintering temperature which are adequate for medical uses. Additionally, these results open the possibility of applying this methodology to synthesize HAp for other different applications. For example, non-medical applications of HAp include packing media for column chromatography, gas sensors, catalyst and host materials; where each application requires different and specific characteristics of HAp.

## 4. Conclusion

Wet precipitation synthesis of HAp powders was successfully performed, and the results obtained indicated that the morphological, crystalline and chemical characteristics of the HAp have a strong dependency on synthesis parameters, mainly the pH value during the synthesis and the sintering temperature. For samples at pH 9, spherical particles around 30–50 nm were observed. At pH 10, HAp flakes approximately 150 nm were identified and at pH 11 resulted in a combination of HAp rods, particle and flakes. Furthermore, the sintering temperature plays a crucial role in the morphology of the HAp nanoparticles. Irrespective of the pH values, as the sintering temperature increases, an effect of densification was found which results in the gathering of those nanostructures synthesized at room temperature and resulted in a flake-like interconnected HAp grain which is an indication of coarsening of the HAp particle size. The TEM analysis confirms the formation of rod- and flake-like HAp structures. XRD analysis revealed the formation of HAp and monetite phases with crystallite size ranging from 20 to 56 nm. It was noted that an increment of the pH leads to a decrease in the monetite and carbonate products, but also a widened Ca/P ratio with an optimum for pH 10. The FTIR analysis showed the presence of the stretching and bending vibrational modes of carbonate, phosphate, water molecule and hydroxyl ion groups. The FTIR analysis also demonstrates the effect of pH by the removal of carbonate vibrational modes resulting in pure HAp phase powders. From the obtained results, we can conclude that an appropriate combination of pH and sintering temperature allow controlling the morphology, crystallinity and chemical characteristics of the HAp for a specific application as biomaterial.

## Supplementary Material

XRD Spectra

## Supplementary Material

FTIR spectra

## Supplementary Material

Crystallite size

## Data accessibility

All the data files of XRD spectra, crystallite size and FTIR spectra reported in this article have been uploaded as part of the electronic supplementary material.

## References

[1] Sadat-Shojai M, Khorasani M-T, Dinpanah-Khoshdargi E, Jamshidi A. 2013 Synthesis methods for nanosized hydroxyapatite in diverse structures. Acta Biomater. 9, 7591–7621. (10.1016/j.actbio.2013.04.012) 23583646

[2] Silver FH, Christiansen DL 1999 Introduction to biomaterials science and biocompatibility. In Biomaterials science and biocompatibility, pp. 1–26. New York, NY: Springer.

[3] Kolmas J, Krukowski S, Laskus A, Jurkitewicz M 2016 Synthetic hydroxyapatite in pharmaceutical applications. Ceram. Int. 42, 2472–2487. (10.1016/j.ceramint.2015.10.048)

[4] Ferraz MP, Monteiro FJ, Manuel CM 2004 Hydroxyapatite nanoparticles: a review of preparation methodologies. J. Appl. Biomater. 2, 74–80. 20803440

[5] Pramanik S, Agarwal AK, Rai KN, Garg A 2007 Development of high strength hydroxyapatite by solid-state-sintering process. Ceram. Int. 33, 419–426. (10.1016/j.ceramint.2005.10.025)

[6] Rodríguez-Lugo V, Camacho-Bragado GA, Castaño VM 2003 Morphological and compositional changes on sand dollar biomaterials induced by heat treatments. Mater. Manuf. Process. 18, 67–78. (10.1081/AMP-120017589)

[7] Rodríguez LV, Castaño VM, Rubio-Rosas E 2016 Biomimetic growth of hydroxylapatite on SiO_2_–PMMA hybrid coatings. Mater. Lett. 184, 265–268. (10.1016/j.matlet.2016.08.068)

[8] Rodríguez-Lugo V, Ascencio JA, Angeles-Chavez C, Camacho-Bragado A, Castaño VM 2001 Controlled hydrothermal production of hydroxylapatite from marine skeletons. Mater. Technol. 16, 97–103. (10.1080/10667857.2001.11752918)

[9] Ascencio J, Rodríguez-Lugo V, Angeles C, Santamaría T, Castaño V 2002 Theoretical analysis of hydroxylapatite and its main precursors by quantum mechanics and HREM image simulation. Comput. Mater. Sci. 25, 413–426. (10.1016/S0927-0256(02)00243-4)

[10] Sáenz A, Montero ML, Mondragón G, Rodríguez-Lugo V, Castaño VM 2003 Effect of pH on the precipitation of hydroxyapatite on silica gels. Mater. Res. Innov. 7, 68–73. (10.1007/s10019-003-0230-x)

[11] Rodríguez-Lugo V, Angeles-Chavez C, Hernandez M 2003 Synthesis of hydroxylapatite from sand dollar and β-tricalcium phosphate by solid-state method. Mater. Manuf. Process. 18, 903–913. (10.1081/AMP-120025078)

[12] Recillas S, Mondragón-Galicia G, Rodríguez-Lugo V, Castaño VM 2003 Growth of calcium phosphate onto chemically-functionalized cottons. Des. Monomers Polym. 6, 383–398. (10.1163/156855503771816840)

[13] Ślósarczyk A, Paszkiewicz Z, Paluszkiewicz C 2005 FTIR and XRD evaluation of carbonated hydroxyapatite powders synthesized by wet methods. J. Mol. Struct. 744–747, 657–661. (10.1016/j.molstruc.2004.11.078)

[14] Rodríguez-Lugo V, Angeles-Chavez C, Mondragon G, Recillas-Gispert S, Castaño VM 2005 Synthesis and structural characterization of hydroxyapatite obtained from CaO and CaHP0_4_ by a hydrothermal method. Mater. Res. Innov. 9, 20–22. (10.1080/14328917.2005.11784875)

[15] Tang XL, Xiao XF, Liu RF 2005 Structural characterization of silicon-substituted hydroxyapatite synthesized by a hydrothermal method. Mater. Lett. 59, 60 (10.1016/j.matlet.2005.06.060)

[16] Yoon SY, Park YM, Park SS, Stevens R, Park HC 2005 Synthesis of hydroxyapatite whiskers by hydrolysis of α-tricalcium phosphate using microwave heating. Mater. Chem. Phys. 91, 48–53. (10.1016/j.matchemphys.2004.10.049)

[17] Rodríguez-Lugo V, Angeles C, Isla A, Castano VM 2015 Effect of bio-calcium oxide on the morphology of hydroxyapatite. Int. J. Basic Appl. Sci. 4, 395–403. (doi:10.14419/ijbas.v4i4.5240)

[18] Wei G, Ma PX 2004 Structure and properties of nano-hydroxyapatite/polymer composite scaffolds for bone tissue engineering. Biomaterials 25, 4749–4757. (10.1016/j.biomaterials.2003.12.005) 15120521

[19] Murugan R, Ramakrishna S 2004 Bioresorbable composite bone paste using polysaccharide based nano hydroxyapatite. Biomaterials 25, 3829–3835. (10.1016/j.biomaterials.2003.10.016) 15020158

[20] Wang H, Li Y, Zuo Y, Li J, Ma S, Cheng L 2007 Biocompatibility and osteogenesis of biomimetic nano-hydroxyapatite/polyamide composite scaffolds for bone tissue engineering. Biomaterials 28, 3338–3348. (10.1016/j.biomaterials.2007.04.014) 17481726

[21] Wang X, Li Y, Wei J, de Groot K 2002 Development of biomimetic nano-hydroxyapatite/poly(hexamethylene adipamide) composites. Biomaterials 23, 4787–4791. (10.1016/S0142-9612(02)00229-6) 12361617

[22] Sugimoto T 2000 Fine particles: synthesis, characterization, and mechanisms of growth. New York, NY: Marcel Dekker.

[23] Mishra VK, Bhattacharjee BN, Kumar D, Rai SB, Parkash O 2016 Effect of a chelating agent at different pH on the spectroscopic and structural properties of microwave derived hydroxyapatite nanoparticles: a bone mimetic material. New J. Chem. 40, 5432–5441. (10.1039/c5nj03322e)

[24] Rajkumar M, Sundaram NM, Rajendran V 2011 Preparation of size controlled, stoichiometric and bioresorbable hydroxyapatite nanorod by varying initial pH, Ca/P ratio and sintering temperature. Dig. J. Nanomater. Biostruct. 6, 169–179.

[25] Cüneyt TA, Korkusuz F, Timuçin M, Akkaş N 1997 An investigation of the chemical synthesis and high-temperature sintering behaviour of calcium hydroxyapatite (HA) and tricalcium phosphate (TCP) bioceramics. J. Mater. Sci. Mater. Med. 8, 91–96. (10.1023/A:1018506800033) 15348776

[26] Pang YX, Bao X 2003 Influence of temperature, ripening time and calcination on the morphology and crystallinity of hydroxyapatite nanoparticles. J. Eur. Ceram. Soc. 23, 1697–1704. (10.1016/S0955-2219(02)00413-2)

[27] Mostafa NY 2005 Characterization, thermal stability and sintering of hydroxyapatite powders prepared by different routes. Mater. Chem. Phys. 94, 333–341. (10.1016/j.matchemphys.2005.05.011)

[28] Kothapalli C, Wei M, Vasiliev A, Shaw MT 2004 Influence of temperature and concentration on the sintering behavior and mechanical properties of hydroxyapatite. Acta Mater. 52, 5655–5663. (10.1016/j.actamat.2004.08.027)

[29] Cao Y, Yang B, Gao C, Feng P, Shuai C 2015 Laser sintering of nano 13–93 glass scaffolds: microstructure, mechanical properties and bioactivity. Sci. Sinter. 47, 31–39. (10.2298/SOS1501031C)

[30] Radha G, Balakumar S, Venkatesan B, Vellaichamy E 2015 Evaluation of hemocompatibility and in vitro immersion on microwave-assisted hydroxyapatite–alumina nanocomposites. Mater. Sci. Eng. C 50, 143–150. (10.1016/J.MSEC.2015.01.054) 25746256

[31] Liu Y, Hou D, Wang G 2004 A simple wet chemical synthesis and characterization of hydroxyapatite nanorods. Mater. Chem. Phys. 86, 69–73. (10.1016/j.matchemphys.2004.02.009)

[32] Rezwan K, Chen QZ, Blaker JJ, Boccaccini AR 2006 Biodegradable and bioactive porous polymer/inorganic composite scaffolds for bone tissue engineering. Biomaterials 27, 3413–3431. (10.1016/j.biomaterials.2006.01.039) 16504284

[33] Kim S-S, Sun Park M, Jeon O, Yong Choi C, Kim B-S 2006 Poly(lactide-co-glycolide)/hydroxyapatite composite scaffolds for bone tissue engineering. Biomaterials 27, 1399–1409. (10.1016/j.biomaterials.2005.08.016) 16169074

[34] Abidi SSA, Murtaza Q 2014 Synthesis and characterization of nano-hydroxyapatite powder using wet chemical precipitation reaction. J. Mater. Sci. Technol. 30, 307–310. (10.1016/j.jmst.2013.10.011)

[35] Rhee S-H 2002 Synthesis of hydroxyapatite via mechanochemical treatment. Biomaterials 23, 1147–1152. (10.1016/S0142-9612(01)00229-0) 11791918

[36] Fathi MH, Hanifi A, Mortazavi V 2008 Preparation and bioactivity evaluation of bone-like hydroxyapatite nanopowder. J. Mater. Process. Technol. 202, 536–542. (10.1016/j.jmatprotec.2007.10.004)

[37] Raynaud S, Champion E, Bernache-Assollant D, Thomas P 2002 Calcium phosphate apatites with variable Ca/P atomic ratio I. Synthesis, characterisation and thermal stability of powders. Biomaterials 23, 1065–1072. (10.1016/S0142-9612(01)00218-6) 11791909

[38] Raynaud S, Champion E, Bernache-Assollant D 2002 Calcium phosphate apatites with variable Ca/P atomic ratio II. Calcination and sintering. Biomaterials 23, 1073–1080. (10.1016/S0142-9612(01)00219-8) 11791910

[39] Venkateswarlu K, Chandra Bose A, Rameshbabu N 2010 X-ray peak broadening studies of nanocrystalline hydroxyapatite by Williamson Hall analysis. Phys. B Condens. Matter 405, 4256–4261. (10.1016/j.physb.2010.07.020)

[40] Jagodzinski H, Klug HP, Alexander LE 1975 X-ray diffraction procedures for polycrystalline and amorphous materials. New York, NY: Wiley.

[41] Elhendawi H, Felfel RM, Abd El-Hady BM, Reicha FM 2014 Effect of synthesis temperature on the crystallization and growth of in situ prepared nanohydroxyapatite in chitosan matrix. ISRN Biomater. 2014, 1–8. (10.1155/2014/897468)

[42] Ooi CY, Hamdi M, Ramesh S 2007 Properties of hydroxyapatite produced by annealing of bovine bone. Ceram. Int. 33, 1171–1177. (10.1016/j.ceramint.2006.04.001)

[43] Wang P, Li C, Gong H, Jiang X, Wang H, Li K 2010 Effects of synthesis conditions on the morphology of hydroxyapatite nanoparticles produced by wet chemical process. Powder Technol. 203, 315–321. (10.1016/j.powtec.2010.05.023)

[44] Thanh NTK, Maclean N, Mahiddine S 2014 Mechanisms of nucleation and growth of nanoparticles in solution. Chem. Rev. 114, 7610–7630. (10.1021/cr400544s) 25003956

[45] Maisara SM, Arsad PML 2011 Synthesis and characterization of hydroxyapatite nanoparticles and β-TCP particles. In *2nd Int. Conf. on Biotechnology and Food Science*, pp. 184–188 Singapore: IACSIT Press.

[46] Wang Y, Ren X, Ma X, Su W, Zhang Y, Sun X, Li X 2015 Alginate-intervened hydrothermal synthesis of hydroxyapatite nanocrystals with nanopores. Cryst. Growth Des. 15, 1949–1956. (10.1021/acs.cgd.5b00113)

[47] Lai W, Chen C, Ren X, Lee IS, Jiang G, Kong X 2016 Hydrothermal fabrication of porous hollow hydroxyapatite microspheres for a drug delivery system. Mater. Sci. Eng. C 62, 166–172. (10.1016/j.msec.2016.01.055) 26952411

[48] An L, Li W, Xu Y, Zeng D, Cheng Y, Wang G 2015 Controlled additive-free hydrothermal synthesis and characterization of uniform hydroxyapatite nanobelts. Ceram. Int. 42, 3104–3112. (10.1016/j.ceramint.2015.10.099)

[49] Joschek S, Nies B, Krotz R, Göpferich A 2000 Chemical and physicochemical characterization of porous hydroxyapatite ceramics made of natural bone. Biomaterials 21, 1645–1658. (10.1016/S0142-9612(00)00036-3) 10905406

[50] Walters MA, Leung YC, Blumenthal NC, LeGeros RZ, Konsker KA 1990 A Raman and infrared spectroscopic investigation of biological hydroxyapatite. J. Inorg. Biochem. 39, 193–200. (10.1016/0162-0134(90)84002-7) 2168470

[51] Hu Y, Miao X 2004 Comparison of hydroxyapatite ceramics and hydroxyapatite/borosilicate glass composites prepared by slip casting. Ceram. Int. 30, 1787–1791. (10.1016/j.ceramint.2003.12.119)

[52] Murugan R, Ramakrishna S 2004 Effect of zirconia on the formation of calcium phosphate bioceramics under microwave irradiation. Mater. Lett. 58, 230–234. (10.1016/S0167-577X(03)00451-8)

[53] Ahn ES, Gleason NJ, Nakahira A, Ying JY 2001 Nanostructure processing of hydroxyapatite-based bioceramics. Nano Lett. 1, 149–153. (10.1021/nl0055299)

[54] Rusu VM, Ng CH, Wilke M, Tiersch B, Fratzl P, Peter MG 2005 Size-controlled hydroxyapatite nanoparticles as self-organized organic-inorganic composite materials. Biomaterials 26, 5414–5426. (10.1016/j.biomaterials.2005.01.051) 15814140

[55] Chaopanich P, Siriphannon P 2015 Facile refluxing synthesis of hydroxyapatite nanoparticles. Aust. J. Chem. 68, 1293–1298. (10.1071/CH14642)

[56] Zhang H, Darvell BW 2011 Morphology and structural characteristics of hydroxyapatite whiskers: effect of the initial Ca concentration, Ca/P ratio and pH. Acta Biomater. 7, 2960–2968. (10.1016/j.actbio.2011.03.020) 21421085

[57] Peng H, Wang J, Lv S, Wen J, Chen J 2015 Synthesis and characterization of hydroxyapatite nanoparticles prepared by a high-gravity precipitation method. 41, 14 340–14 349. (10.1016/j.ceramint.2015.07.067)

[58] Vázquez-Hernández F, Mendoza-Barrera C, Altuzar V, Meléndez-Lira M, Santana-Aranda MA, Olvera M 2010 Synthesis and characterization of hydroxyapatite nanoparticles and their application in protein adsorption. Mater. Sci. Eng. B 174, 290–295. (10.1016/j.mseb.2010.03.011)

[59] Muralithran G, Ramesh S 2000 The effects of sintering temperature on the properties of hydroxyapatite. Ceram. Int. 26, 221–230. (10.1016/S0272-8842(99)00046-2)

[60] Van Landuyt P, Li F, Keustermans JP, Streydio JM, Delannay F, Munting E 1995 The influence of high sintering temperatures on the mechanical properties of hydroxylapatite. J. Mater. Sci. Mater. Med. 6, 8–13. (10.1007/BF00121239)

